# A small molecule drug promoting miRNA processing induces alternative splicing of *MdmX* transcript and rescues p53 activity in human cancer cells overexpressing MdmX protein

**DOI:** 10.1371/journal.pone.0185801

**Published:** 2017-10-03

**Authors:** Georgios Valianatos, Barbora Valcikova, Katerina Growkova, Amandine Verlande, Jitka Mlcochova, Lenka Radova, Monika Stetkova, Michaela Vyhnakova, Ondrej Slaby, Stjepan Uldrijan

**Affiliations:** 1 Department of Biology, Faculty of Medicine, Masaryk University, Brno, Czech Republic; 2 International Clinical Research Center, St. Anne's University Hospital, Brno, Czech Republic; 3 Central European Institute of Technology, Masaryk University, Brno, Czech Republic; Augusta University, UNITED STATES

## Abstract

MdmX overexpression contributes to the development of cancer by inhibiting tumor suppressor p53. A switch in the alternative splicing of *MdmX* transcript, leading to the inclusion of exon 6, has been identified as the primary mechanism responsible for increased MdmX protein levels in human cancers, including melanoma. However, there are no approved drugs, which could translate these new findings into clinical applications. We analyzed the anti-melanoma activity of enoxacin, a fluoroquinolone antibiotic inhibiting the growth of some human cancers *in vitro* and *in vivo* by promoting miRNA maturation. We found that enoxacin inhibited the growth and viability of human melanoma cell lines much stronger than a structurally related fluoroquinolone ofloxacin, which only weakly modulates miRNA processing. A microarray analysis identified a set of miRNAs significantly dysregulated in enoxacin-treated A375 melanoma cells. They had the potential to target multiple signaling pathways required for cancer cell growth, among them the RNA splicing. Recent studies showed that interfering with cellular splicing machinery can result in MdmX downregulation in cancer cells. We, therefore, hypothesized that enoxacin could, by modulating miRNAs targeting splicing machinery, activate p53 in melanoma cells overexpressing MdmX. We found that enoxacin and ciprofloxacin, a related fluoroquinolone capable of promoting microRNA processing, but not ofloxacin, strongly activated wild type p53-dependent transcription in A375 melanoma without causing significant DNA damage. On the molecular level, the drugs promoted *MdmX* exon 6 skipping, leading to a dose-dependent downregulation of MdmX. Not only in melanoma, but also in MCF7 breast carcinoma and A2780 ovarian carcinoma cells overexpressing MdmX.

Together, our results suggest that some clinically approved fluoroquinolones could potentially be repurposed as activators of p53 tumor suppressor in cancers overexpressing MdmX oncoprotein and that p53 activation might contribute to the previously reported activity of enoxacin towards human cancer cells.

## Introduction

Several key tumor suppressor pathways are inactivated during the development of most, if not all, human cancers. A prime example of such frequently inactivated tumor suppressor is p53, an important regulator of cellular responses to stress stimuli, such as hypoxia, DNA damage, oncogene activation, telomere shortening or metabolic stress [1]. Its function is commonly lost in cancers by mutations in the *p53* gene or by overexpression of cellular inhibitory proteins Mdm2 and MdmX (also known as Mdm4 or HdmX) that cooperate to inhibit p53-mediated transcription by binding to its transactivation domain and by targeting p53 for proteasomal degradation [2]. Mdm2 and MdmX are essential for keeping p53 activity low in normal untransformed and unstressed cells, but their overexpression is estimated to contribute to the loss of p53 activity in up to two million cancer cases worldwide every year [3]. MdmX has been identified as a key therapeutic target in malignant melanoma, with MdmX protein levels increased in over sixty per cent of tumors [4]. There are other cancers with a known or suspected role of MdmX overexpression in tumor development or progression, such as, for example, retinoblastoma [5,6], breast carcinoma [7,8], or chronic lymphocytic leukemia (CLL) [9].

A recent study showed that a switch in alternative splicing of *MdmX* transcript is primarily responsible for increased MdmX protein levels in cancer cells, including melanoma [10]. Normal adult tissues produce *MdmX-S* isoform as a result of exon 6 skipping that is targeted by the nonsense-mediated mRNA decay pathway, while enhanced exon 6 inclusion leads to the expression of full-length *MdmX* in a significant number of human cancers. Intriguingly, antisense oligonucleotide–mediated skipping of exon 6 decreased MdmX abundance, inhibited melanoma growth, and enhanced sensitivity to BRAF inhibitors in human melanoma cell lines and melanoma patient–derived xenografts [10]. Other studies also suggested the importance of alternative splicing of *MdmX* as the ratio between the short *MdmX-S* isoform and the full-length *MdmX* transcript strongly correlated with MdmX protein levels and could serve as a prognostic marker in osteosarcoma, breast carcinoma and CLL [11–13]. Unfortunately, currently, there are no small molecule compounds approved for use in humans or advanced in clinical testing, which could help to translate these recent findings into clinical therapeutic use quickly.

MicroRNAs (miRNAs) are small non-coding RNAs that can regulate gene expression by inducing cleavage of their target mRNAs or by inhibiting their translation [14]. Human cancers commonly exhibit global downregulation of microRNA expression, and restoration of normal microRNA levels might, therefore, represent an attractive approach in cancer therapy [15–17]. Small molecule fluoroquinolone drug enoxacin was able to effectively restore TARBP2-mediated miRNA processing in a panel of cancer cell lines from several common malignancies and had a cancer-specific inhibitory effect on cell growth both *in vitro* and *in vivo* [18,19]. In this study, we analyzed the response of malignant melanoma cells to enoxacin and other clinically approved fluoroquinolones. We present data suggesting that enoxacin and ciprofloxacin can efficiently promote *MdmX* exon 6 skipping, downregulate MdmX protein levels, and activate the p53 pathway not only in melanoma but also in other types of cancer overexpressing MdmX.

## Materials and methods

### Cell culture and treatments

The human cancer cell lines A375, Mel-Juso, Mel-Ho, IPC298, H1299, A2780, and MCF7 (obtained from ECACC, DSMZ, and ATCC) were cultured at 37°C and 5% CO_2_ in RPMI-1640 medium (Sigma-Aldrich), supplemented with 10% fetal bovine serum, 2mM L-glutamine, 100 IU/mL penicillin and 100 μg/mL streptomycin. The AmpFLSTR® Identifiler® PCR Amplification Kit (Life Technologies) was used to verify the identity of the cell lines. The MCF7-DDp53 and A375-DDp53 cell lines stably overexpressing a dominant-negative truncated mouse p53 protein have been described previously [20,21]. The fluoroquinolone antibiotics used in this study (enoxacin, ciprofloxacin, and ofloxacin) were purchased from Sigma-Aldrich. DNA-damaging drugs doxorubicin and etoposide were purchased from Sigma-Aldrich and Enzo Life Sciences, respectively.

### Analysis of *MdmX* alternative splicing

Total RNA was isolated using RNA Blue (Top-Bio) according to the manufacturer’s instructions. RNA quantity was assessed on NanoDrop 1000 (Thermo Scientific). Consequently, 1 μg of RNA was transcribed into cDNA in the total volume of 20 μl with oligo-(dT)_15_ primer and Transcriptor Reverse Transcriptase (Roche). Semi-quantitative was performed PCR performed using MyFi Mix^TM^ (Bioline), and PCR products were run on 2.5% agarose gel and visualized with GoodView nucleic acid stain (Ecoli). PCR conditions, including cycle number and annealing temperature, were optimized for each set of primers. The PCR reactions were carried out in a total volume of 20 μl and consisted of 35 cycles of 25 s denaturation step at 95°C, 30 s primer annealing (49°C for MdmX primers 1 and 58°C for MdmX primers 2), and 20 s polymerization step at 72°C. The following sets of PCR primers were used (primer sequences in 5’-3‘ orientation): *MdmX* primers 1 (F1 *GCAGTTTCTTCACTACCA*, R1 *AGCCTAGATGTTTCATCTTG*) [22], *MdmX* primers 2 (F2 *TGTGGTGGAGATCTTTTGGG*, R2 *GCAGTGTGGGGATATCGT*) [10], and *GAPDH* primers (forward *AATCCCATCACCATCTTCC*, reverse *ATGAGTCCTTCCACGATACC*) [11]. PCR amplification of *GAPDH* served as a loading control. To determine the nature of the additional band in RT-PCR reactions with *MdmX* primers 2, A375 and MCF7 cells were transfected with a mixture of MdmX-specific siRNAs (siRNA1 *5'-AUG CAU ACA UUC UAG AGA ATT-3'*, siRNA2 *5'-GGA AGG AUU GGU AUU CAG ATT-3'*) using X-tremeGENE siRNA Transfection Reagent (Sigma-Aldrich) and were analyzed 24 hours later.

### Western blotting

Cells were washed with PBS and lysed in 2x Laemli Sample buffer. The lysates were boiled, separated by SDS-PAGE and transferred to PVDF membranes (Merck Millipore). The membranes were blocked in 5% dry milk in TBS-Tween for 1 h at room temperature and incubated overnight at 4°C with primary antibodies. Anti-MdmX mouse mAb (clone 8C6) and anti-Mdm2 mouse mAb (clone IF-2), anti-P-p53 (S15) mouse mAb (clone 16G8), and anti-H2A.X phospho (Ser139) (clone 2F3) were purchased from Merck-Millipore, Cell Signaling Technology and BioLegend, respectively. Anti-Mdm2 mouse monoclonal antibodies 2A9 and 2A10, anti-PCNA mouse mAb PC-10, and anti-p53 mouse mAb DO-1 were kindly provided by Dr. Borek Vojtesek, Masaryk Memorial Cancer Institute, Brno, Czech Republic. After washing in 1% dry milk in TBS-Tween for 3x 10 minutes, the membranes were incubated for 1 hour at room temperature with the horseradish peroxidase-conjugated donkey anti-mouse secondary antibody (Santa Cruz Biotechnology). The proteins of interest were visualized using ECL substrate (Thermo Scientific) together with chemiluminescence detection system G:BOX (Syngene).

### Analysis of p53 transcriptional activity

Wild type p53-expressing melanoma cell lines A375 and Mel-Juso were transfected with the expression vector pGL4.38 [luc2P/p53 RE/Hygro] using TurboFect (Thermo Scientific), following the manufacturer’s instructions. Hygromycin was added to the growth media (100 μg/ml) twenty-four hours post-transfection, and stable transfectants were selected for ten days. To determine the p53 activity, the stably transfected cells were lysed 24 h post-treatment in Pierce Luciferase Cell Lysis Buffer (Thermo Scientific) for 15 min on ice. Total protein concentrations in lysates were determined using Bradford Reagent (Bio-Rad), and luciferase activity was measured using Pierce Firefly Luciferase Glow Assay Kit (Thermo Scientific) per the manufacturer’s instructions. Transient transfections of the pGL4.38 [luc2P/p53 RE/Hygro] construct into wild type and DDp53-expressing A375 and MCF7 cells were performed to confirm the inhibitory effect of the DDp53 mini-protein on p53 activity. Cells were treated with enoxacin or doxorubicin 24 hours post-transfection and luciferase activity in cell lysates was determined 24 hours later.

### Cell viability

Cells were seeded at the concentration of 7*10^4^ cells/ml and 24 h later treated with enoxacin in concentrations 25, 50, 75, and 100 μg/ml. After three-day incubation with the drug, cells were harvested, washed in cold PBS, and PI (1 μg/ml, Sigma-Aldrich) was added. The percentage of dead (PI positive) cells was measured using Attune Acoustic Focusing Cytometer (Applied Biosystems) at 620 nm emission wavelength. A total number of 10,000 cells per sample was analyzed.

### Cell proliferation

Cells were grown in standard growth medium in the presence of enoxacin (50 μg/ml) or 0.2% NaOH (vehicle, negative control). Relative cell growth was determined using standard MTT assay after 1, 2, 3, 4, or 5 days in culture in the presence of the drug.

### Quantitative real-time PCR

Quantitative RT-PCR was performed using LightCycler 480 Instrument (Roche) in a final reaction volume of 10 μl. Each reaction included 1 μl of cDNA template, 5 μl of 2x TaqMan Gene Expression Master Mix (Applied Biosystems), 0.5 μl of the respective primer/probe set labeled with FAM/MGB and 0.5 μl of primer/probe set labeled with VIC/MGB (for GAPDH) and 3 μl of water. Amplification of housekeeping gene GAPDH (#4310884E, Thermo Fisher) was used for normalization. The PCR was carried out with dual color hydrolysis probe/UPL probe as follows: 10 min pre-incubation at 95°C and 45 cycles of amplification: 10 s at 95°C, 30 s at 60°C and 1 s at 72°C. Assays were performed in triplicates, and the mean Ct value of each triplicate was used for analysis by the 2(- Delta Delta Ct) method. The following TaqMan Gene Expression Assays (Thermo Fisher Scientific) were used: MdmX (Hs00967238_m1), BAX (Hs00180269_m1), BBC3/PUMA (Hs00248075_m1), p21/CDKN1A (Hs00355782_m1), GADD45 (Hs00169255_m1), Mdm2 (Hs01066930_m1), HPRT1 (HGPRT) Endogenous Control (#4333768F).

### Analysis of miRNA expression

Total RNA was extracted with mirVana™ miRNA Isolation Kit (Ambion), following the manufacturer's instructions. Total RNA concentration and purity were controlled by UV spectrophotometry using Nanodrop ND-1000 (Thermo Scientific). For whole-genome miRNA expression profiling, total RNA, including the small RNA fraction, was used and analyzed with Affymetrix GeneChip miRNA 3.0 arrays (Affymetrix) containing 5607 probe sets for human small RNAs. Out of these probe sets the 1733 probe sets of mature human miRNAs were filtered. These probe sets guarantee 100% coverage of all mature human miRNAs in miRBase v.17 (April 2011). Probe sets that were deleted in a more recent version of miRBase were excluded for analysis. All steps of the procedure were performed according to the Affymetrix standardized protocol for miRNA 3.0 arrays. Briefly, 500 ng of total RNA was poly-A tailed, followed by labeling using the FlashTag Biotin HSR RNA Labeling Kit (Genisphere). A hybridization cocktail was added to the biotin-labeled RNA sample and heated to 99°C for 5 minutes and then to 45°C for another 5 minutes. This mixture was injected into an Affymetrix miRNA 3.0 array, and hybridization was performed under rotation at 48°C for 16 hours. After washing and staining steps using the Affymetrix Fluidics Station, the arrays were scanned on the Affymetrix 3000 GeneScanner. Intensity values for each probe cell (.cel file) were calculated using Affymetrix GeneChip Command Console (AGCC). Quality control of the microarray was performed with the Affymetrix miRNA QC Tool, version 1.1.1.0. The Affymetrix raw data (.cel files) were normalized using the robust multichip average (RMA) algorithm from 'oligo' Bioconductor package [23] in R version 3.0.1 [24]. The LIMMA package [25] was used to identify differentially expressed miRNA probe sets between different groups. Obtained p-values were adjusted for multiple testing using the Benjamini-Hochberg correction. Annotations of miRNA probe sets were derived from the Sanger miRBase database v.20 (June 2013, http://mirbase.org).

## Results

### Enoxacin inhibits growth of melanoma cells

**Fig 1:**
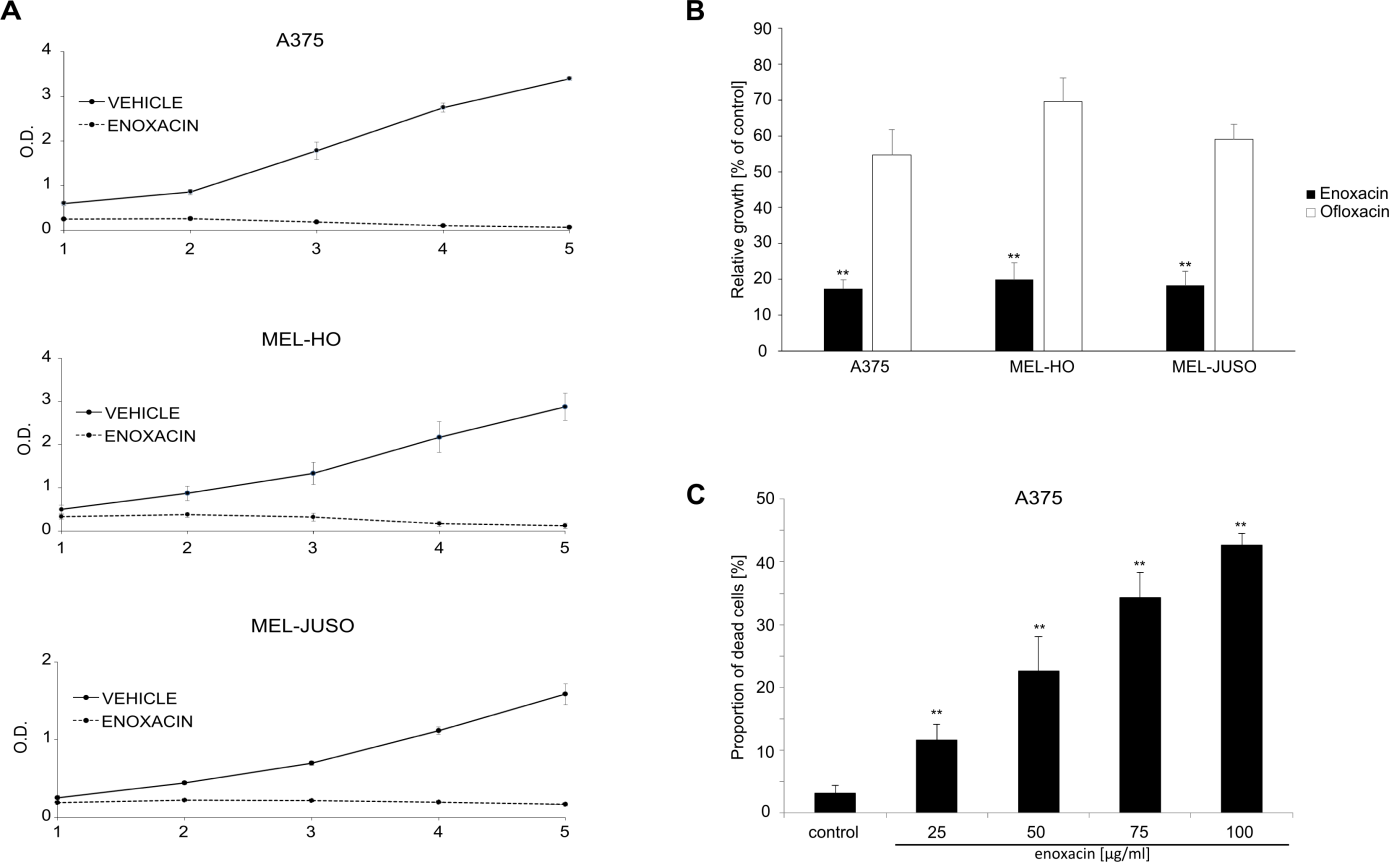
Enoxacin inhibits melanoma cell growth. (**A**) Melanoma cell lines were grown in the presence of enoxacin (50 μg/ml) or 0.2% NaOH (vehicle, negative control). Relative cell growth was determined using standard MTT assay after 1, 2, 3, 4, and 5 days. Results of three independent experiments are presented (means +/- SD). (**B**) Melanoma cell lines were grown in the presence of 0.2% NaOH (control), enoxacin or ofloxacin (both 50 μg/ml) for four days and relative cell growth was determined by MTT assays. Results of three independent experiments (means + standard deviations) are presented. A significant difference between the effect of enoxacin and ofloxacin **P<0.01 (Student’s t-test, two-tailed). (**C**) A375 cells were grown for 3 days in the presence of 0.2% NaOH (control) or enoxacin in concentrations 25, 50, 75, and 100 μg/ml. Cell viability was determined using propidium iodide exclusion assay and flow cytometry. Results of three independent experiments are presented (means + SD). ** P<0.01 (Student’s t-test, two-tailed).

To determine the activity of enoxacin in malignant melanoma, we analyzed the growth of three human melanoma cell lines during five-day treatment with 50 μg/ml (156.09 μM) enoxacin in MTT proliferation assays. This concentration was similar to the active concentrations determined in other cancer types in previous studies [18,19]. Enoxacin strongly inhibited the growth of melanoma cells carrying the most common BRAF kinase oncogenic mutation V600E (Mel-Ho, A375), as well as the proliferation of cells with activating mutations in the NRAS oncogene (Mel-Juso) (Fig 1A). In the next experiment, we took advantage of the finding that closely related fluoroquinolone drugs differ in their ability to promote miRNA maturation [26]. Ofloxacin, which is not capable of efficiently promoting miRNA biogenesis, had a much weaker effect on the growth of melanoma cell cultures than enoxacin in our proliferation assays (both drugs used at 50 μg/ml) (Fig 1B). This result indicated that the ability to modulate miRNA processing might be required for the anti-melanoma activity of fluoroquinolones. Next, we measured A375 melanoma cell viability after three-day incubation with enoxacin concentrations ranging from 25 to 100 μg/ml using a propidium iodide-based flow cytometric viability assay. Results presented in Fig 1C showed a dose-dependent increase in the proportion of dead cells in the population of enoxacin-treated A375 cells and indicated that this drug not only inhibited melanoma cell proliferation but it could also have a negative impact on their viability.

### Enoxacin induces changes in the expression pattern of a large set of mature miRNAs in A375 cells

**Fig 2:**
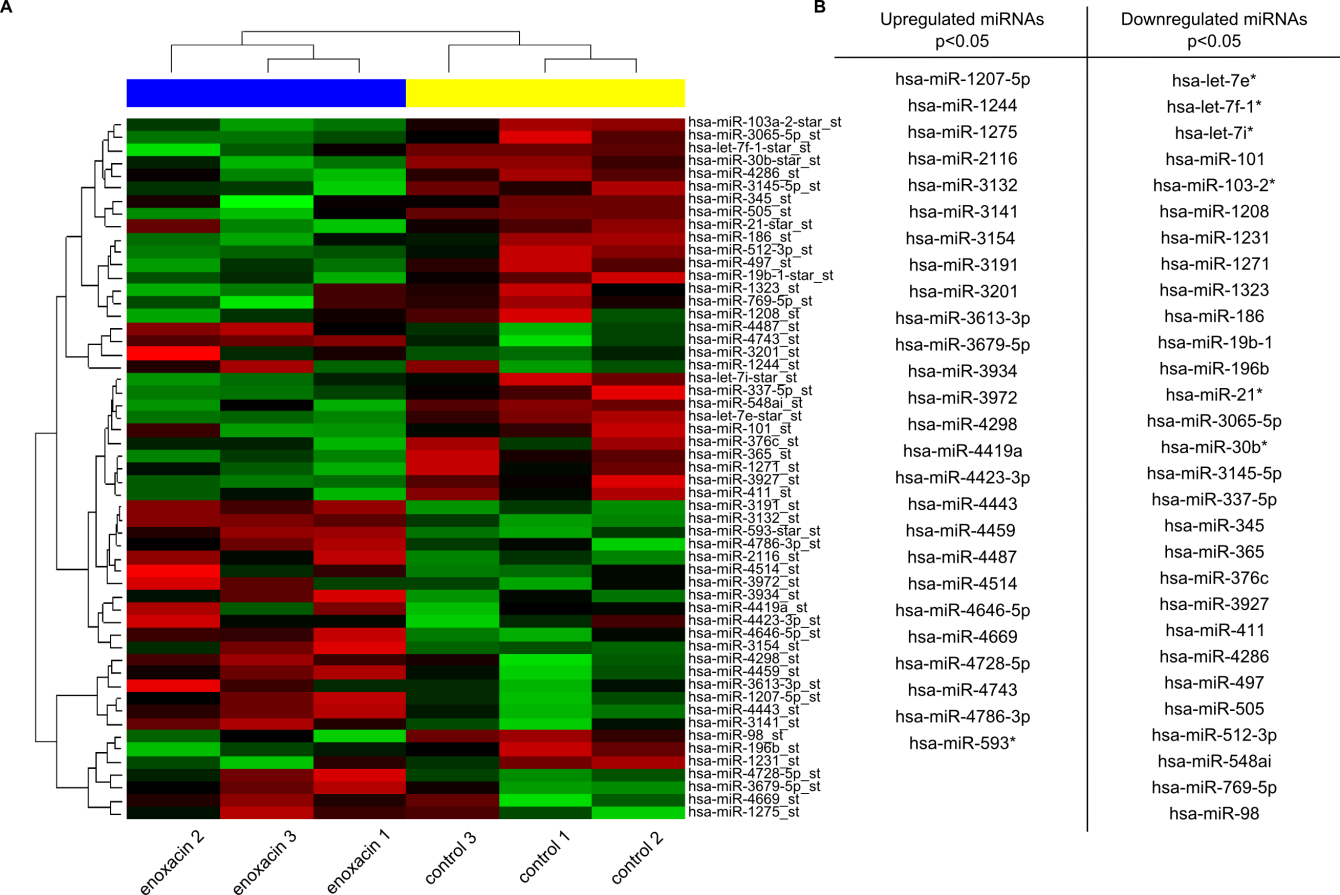
Enoxacin induces changes in the expression pattern of a large set of matured miRNAs in A375 cells. (**A**) miRNA expression profiling of control and enoxacin-treated A375 cells. Total RNA, including the small RNAs, was analyzed with Affymetrix GeneChip miRNA 3.0 arrays. The heat map shows statistically significant changes in the expression of miRNAs in A375 melanoma cells in response to enoxacin treatment (p<0.05; three independent experiments). (**B**) Matured miRNAs identified as significantly up- or down-regulated in A375 melanoma cells in response to enoxacin (p < 0.05).

To identify specific miRNAs that could contribute to growth inhibition in enoxacin-treated melanoma cells, we performed miRNA expression profiling of control and enoxacin-treated A375 cells. Total RNA, including the small RNAs, was analyzed with Affymetrix GeneChip miRNA 3.0 arrays containing 5607 probe sets for small human RNAs. Levels of fifty-five matured miRNAs were identified as significantly changing in response to enoxacin (p < 0.05) (Fig 2A and S1 Table). Of these, twenty-six matured miRNAs were identified as upregulated and twenty-nine as downregulated in response to enoxacin (p < 0.05) (Fig 2B).

To determine the potential targets of the identified miRNAs, aggregation on the geometric mean of the ranks of target lists from multiple sources was applied using the miRNAtap R/Bioconductor package [27]. Targets were aggregated from five most commonly cited prediction algorithms: DIANA [28], Miranda [29], PicTar [30], TargetScan [31], and miRDB [32]. In total, 3924 predicted target genes occurring in at least three sources out of five for each miRNA were annotated with KEGG (Kyoto Encyclopedia of Genes and Genomes) pathway database, and hypergeometric tests were used to estimate the significance of enrichment. This analysis identified 58 cellular pathways potentially targeted by the miRNAs, the majority relevant to cancer (S2 Table). Functional Profile of the selected 3924 genes set at specific Gene Ontology (GO) level (molecular functions, biological processes, and cellular components) was performed (S3 Table). Finally, GO enrichment analysis of the 3924 genes set was applied with the Benjamini-Hochberg multiple comparison correction (S4 Table). Together, these analyses suggested that miRNAs significantly dysregulated in A375 cells in response to enoxacin might modulate a broad range of cellular processes that could all collectively impact on various aspects of cancer cell growth or survival.

### Enoxacin activates wild type p53 in melanoma cells by downregulating MdmX protein levels, not by inducing DNA damage response

Most melanomas retain wild type p53 but overexpress its negative regulator MdmX as a result of a switch in the alternative splicing of the *MdmX* transcript [10]. We were interested in finding out whether enoxacin could, among other cellular processes, regulate the p53 pathway in melanoma cells through its effect on miRNA maturation, as this could contribute to its cytotoxic and antiproliferative effect in melanoma cells. Many miRNAs have been found to regulate the p53-Mdm2-MdmX network directly, and analyses of 3’UTR sequences of *Mdm2* and *MdmX* transcripts suggest that still more might exist [33]. Two of the microRNAs upregulated by enoxacin, miR-3154 and miR-4459, matched those on the miRTarBase 2016 list of miRNAs predicted to target human *MdmX (Mdm4)* transcript (http://miRTarBase.mbc.nctu.edu.tw/ [34]). It might, therefore, be possible that miRNAs induced by enoxacin could directly interfere with *MdmX* expression. Moreover, the analysis of the enriched gene sets identified, among other targets, a relatively large set of genes involved in the regulation of RNA splicing as potentially targeted by miRNAs changing significantly in response to enoxacin (S5 Table). Importantly, in A375 melanoma, *MdmX* splicing was shown to sense and transduce the information about the state of the cellular splicing machinery to the p53 pathway, as RNAi-mediated knock-down of individual components of cell splicing machinery was capable of downregulating MdmX expression and inducing wild type p53-dependent transcriptional activity in the absence of DNA damage [10,35]. Taking these results into account, we hypothesized that by inducing changes in the expression pattern of miRNAs targeting the splicing machinery, enoxacin might be able to modulate *MdmX* splicing, MdmX protein levels and thereby also p53 activity in A375 cells.

**Fig 3:**
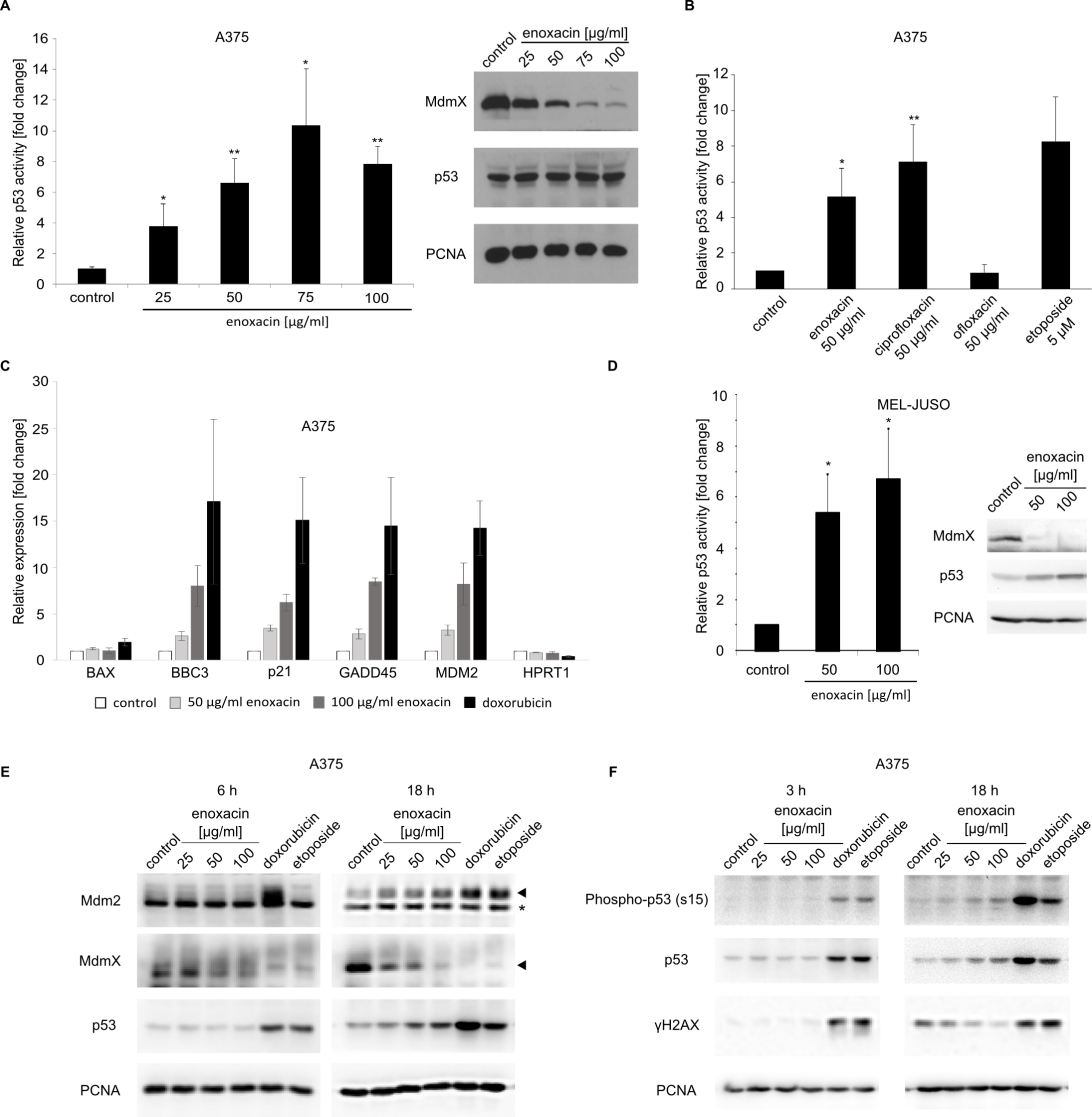
Enoxacin activates p53-dependent transcription in melanoma cells overexpressing MdmX without inducing DNA damage. (**A, left**) A375 cells stably transfected with a p53-responsive luciferase reporter construct were grown for 24 hours in the presence of enoxacin in concentrations 25, 50, 75, and 100 μg/ml. Relative p53 transcriptional activity was determined by measuring the activity of luciferase in cell lysates. Results of three independent experiments are presented (means + SD). * P<0.05, ** P<0.01 (Student’s t-test, two-tailed). (**A, right**) A375 cells were treated with enoxacin for 24 hours, lysed, and protein levels of MdmX and p53 in cell lysates were analyzed by Western blotting. PCNA served as a loading control. (**B**) A375 cells stably transfected with a p53 activity reporter construct were treated with 0.2% NaOH (control), etoposide, and three fluoroquinolone antibiotics, and p53 activity was determined. Results of three independent experiments (means + SD) are presented. A significant difference between control and antibiotic-treated cells * P<0.05, ** P<0.01 (Student’s t-test, two-tailed). (**C**) Quantitative real-time PCR analysis of the expression of selected p53 target genes in A375 cells treated for 24 hours with 50 or 100 μg/ml enoxacin or 0.5 μM doxorubicin. The expression of HPRT1/HGPRT served as an endogenous control. (**D**) Mel-Juso cells stably transfected with a p53-responsive luciferase reporter construct were grown for 24 hours in the presence of enoxacin in concentrations 50 and 100 μg/ml. Relative p53 activity was determined by measuring luciferase activity in cell lysates. Results of three independent experiments are presented (means + SD). * P<0.05, (Student’s t-test, two-tailed). MdmX and p53 protein levels in Mel-Juso cell lysates were analyzed by Western blotting. PCNA served as a loading control. (**E**) (**F**) Western blot analysis of A375 cells treated with increasing concentrations of enoxacin or DNA damaging drugs for the indicated time. A mixture of three different primary monoclonal antibodies recognizing various epitopes in human Mdm2 was used in the Mdm2 panels. * Non-specific band.

To test our hypothesis, A375-p53Luc cells were created by a stable transfection of A375 cells with pGL4.38[luc2P/p53 RE/Hygro], a plasmid construct coding for luciferase under the control of a p53 responsive promoter (Promega). Cells were treated with increasing concentrations of enoxacin (25 to 100 μg/ml) for 24 h, lysed, and luciferase activity was compared between the control and treated cells. Results presented in Fig 3A (left panel) indicated that enoxacin could efficiently activate p53-dependent transcription in A375 melanoma cells. Already at 25 μg/ml enoxacin, we observed an approximately four-fold increase in p53 transcriptional activity after 24 h incubation and the maximal level of p53 activity reached at higher enoxacin concentrations was about ten-fold greater than the control. In line with our hypothesis that enoxacin could be targeting MdmX expression in melanoma, we observed a dose-dependent decrease in MdmX protein levels in enoxacin-treated A375 cells, which could explain the detected changes in p53 activity, despite the absence of significant changes in p53 protein levels (Fig 3A, right panel). While there was an apparent decrease of MdmX levels already at 25 μg/ml, the strongest decrease was seen at enoxacin concentrations 75 and 100 μg/ml. The activation of p53 in our experiments with A375 melanoma correlated with the reported ability of fluoroquinolones to promote miRNA processing. Ciprofloxacin and enoxacin, drugs modulating miRNA biogenesis, activated p53 to an extent similar to that seen in response to a DNA-damaging drug etoposide (Fig 3B). In contrast, ofloxacin, which is not capable of efficiently promoting miRNA biogenesis, did not induce a detectable increase in p53-dependent transcription in our experiments.

To confirm that enoxacin can activate the expression of endogenous p53 targets, we performed quantitative real-time PCR analysis of the expression of selected p53 target genes in A375 cells treated with enoxacin or DNA-damaging drug doxorubicin as a positive control. While there was no significant increase in the expression of *BAX*, the expression of other p53 targets (*BBC3/PUMA*, *p21/CDKN1A*, *GADD45*, and *MDM2*) rose strongly in response to enoxacin (Fig 3C).

In comparison to A375 cells, the NRAS-mutated Mel-Juso melanoma cells expressed significantly lower levels of the MdmX protein and its role in controlling p53 activity in these cells was therefore unclear. However, also in Mel-Juso cells the enoxacin treatment led to a sharp decrease in MdmX protein levels and a relatively small increase in p53 protein levels that was accompanied by a significant activation of p53-dependent transcription (Fig 3D).

Several previous studies have shown that DNA damaging agents can induce alternative splicing of *Mdm2* and *MdmX* transcripts [36,37], and the *MDMX-ALT2* and *MDM2-ALT1* isoforms induced by DNA damage can modulate the transcriptional activity of the stabilized p53 [38]. To exclude the possibility that enoxacin caused DNA damage in melanoma cells, leading to a general DNA damage stress response and possibly also to changes in MdmX expression in our experiments, we compared the response to this drug to the effects on the p53 pathway of DNA damaging chemotherapeutic drugs doxorubicin and etoposide. There was a definite decrease in MdmX protein levels already after 6-hour treatment with DNA damaging drugs or higher enoxacin concentrations, but the maximal response to lower enoxacin concentrations required longer treatment (Fig 3E). To observe possible changes in Mdm2 splicing and expression of smaller Mdm2 isoforms, we used a mixture of three primary antibodies (clones IF-2, 2A9, and 2A10), each targeting a different epitope in the Mdm2 protein. Such experimental design should enable the detection of not only the full length Mdm2 protein but also the major stress-induced splice variant Mdm2-ALT1 [38]. As Mdm2 is not only p53 regulator but also the product of the p53 target gene, we could see a clear Mdm2 induction in response to all treatments at the 18-hour time point. However, we did not detect enhanced induction of short Mdm2 protein isoforms that could indicate changes in the Mdm2 splicing pattern, neither in enoxacin-treated A375 cells nor in response to DNA damage (Fig 3E). An important difference between enoxacin and the DNA damaging agents was the significant stabilization of p53 protein in response to doxorubicin and etoposide already after short 3 or 6 hour treatments, while there was no p53 protein response at these time points in enoxacin-treated A375 cells, despite an apparent effect on the MdmX levels (the 6-hour time point) (Fig 3E and 3F). At the 18-hour time point, a mild increase in the total p53 levels was also observed in the lysates of cells treated with 50 or 100 μg/ml enoxacin, but this increase was weak compared to p53 stabilization induced by much lower concentrations of DNA damaging agents. Moreover, the DNA damaging drugs induced a strong phosphorylation of the p53 protein on serine 15 and the characteristic phosphorylation of serine 139 of histone H2AX, both indicating activation of the DNA damage response pathways. Such response was absent in the enoxacin-treated cells even after 18 hours (Fig 3F), speaking against the induction of a significant amount of DNA damage by the fluoroquinolone. Together, these results suggest that enoxacin did not promote DNA damage-mediated activation of wild type p53 in melanoma cells and that enoxacin-induced changes in *MdmX* expression were DNA damage independent.

Although the doxorubicin treatment alone was already capable of entirely inhibiting MdmX protein expression in A375 cells in our previous experiments (Fig 3E), we decided to also test the possibility that enoxacin and DNA damaging agents might synergize in inducing p53 activity. We treated A375 p53Luc cells with enoxacin and doxorubicin for 18 hours, separately and in combination, and measured p53 activity. Results presented in S1A Fig did not show any additional increase in p53 activity in cells treated with doxorubicin when enoxacin was added.

### Enoxacin promotes *MdmX* exon 6 skipping in cancer cells

As already mentioned, a recent study showed that a switch in alternative splicing of *MdmX* transcript is primarily responsible for increased MdmX protein levels in cancer cells, including A375 melanoma [10]. We hypothesized that enoxacin induced changes in *MdmX* splicing pattern in A375 cells that led to MdmX protein downregulation and p53 activation. Moreover, the fact that enoxacin produced simultaneous shifts in the levels of many miRNAs targeting the expression of multiple genes involved in the regulation of mRNA splicing suggested that its effect on *MdmX* splicing may not be limited to melanoma. Other types of cancer expressing high MdmX protein levels, such as A2780 ovarian carcinoma or MCF7 breast carcinoma, could respond to enoxacin the same way.

**Fig 4:**
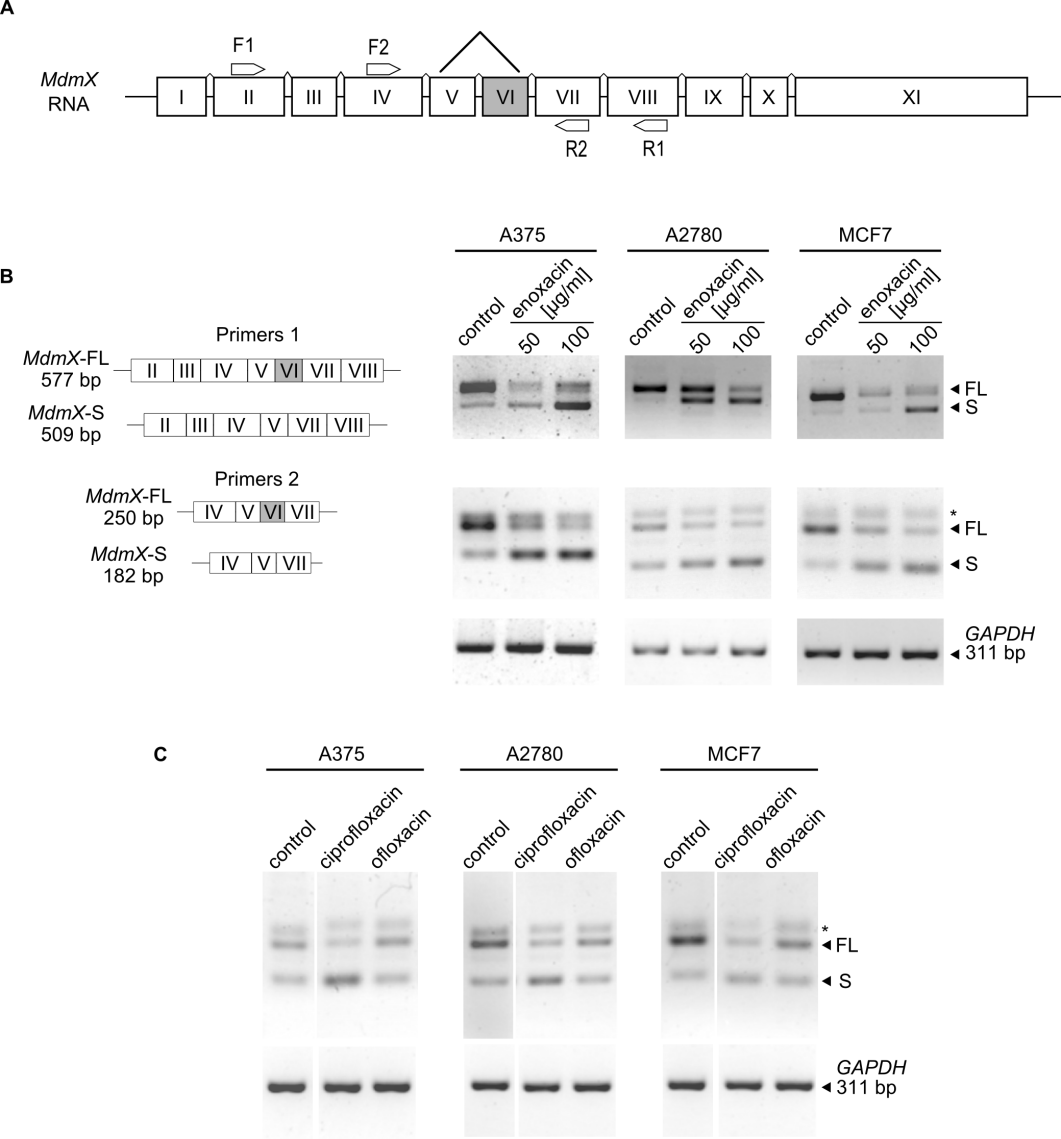
Enoxacin promotes *MdmX* exon 6 skipping in melanoma and non-melanoma cancers overexpressing MdmX. (**A**) Schematic representation of MdmX mRNA exon structure and binding sites for PCR primers used in RT-PCR to determine the presence/absence of exon 6 (VI). (**B, left**) Schematic representation of expected *MdmX* RT-PCR products in the case of presence (*MdmX-FL*) or absence (*MdmX-S*) of exon 6. (**B, right**) RT-PCR analysis of *MdmX* exon 6 skipping in A375, A2780, and MCF7 cells treated with enoxacin (50 and 100 μg/ml) for 24 hours (primers 1 –top, primers 2 –middle, *GAPDH* PCR control–bottom). (**C**) RT-PCR analysis of MdmX exon 6 skipping in A375, A2780 and MCF7 cancer cell lines treated with ciprofloxacin and ofloxacin (both 100 μg/ml) for 24 hours (MdmX primers 2 –top, GAPDH PCR control–bottom). * Non-specific band.

To prove our assumption and to detect the effect of enoxacin on alternative splicing of *MdmX*, we used RT-PCR with two sets of PCR primers (Primers 1: F1-R1 and Primers 2: F2-R2) complementary to different *MdmX* exons (Fig 4A), previously used to detect alternative splicing of *MdmX* in studies by other authors [10,22]. The left panel of Fig 4B depicts the expected sizes of the PCR products in the presence (full length, FL) or absence (short, S) of exon 6 in the *MdmX* transcript. The right panel of Fig 4B shows the results of RT-PCR reactions performed with the two sets of primers using total RNA isolated from vehicle-treated controls and A375, A2780, and MCF7 cells treated for 24 h with two different concentrations of enoxacin. Both PCR reactions showed in all three cell lines an apparent shift in the ratio between the *MdmX-FL* and *MdmX-S* isoforms towards *MdmX-S* in enoxacin-treated cells, suggesting that enoxacin promoted *MdmX* exon 6 skipping in all three different cellular contexts. Similar results were obtained in three independent experiments.

The analysis of RT-PCR results obtained using Primer pair 2 repeatedly showed an additional band above the *MdmX-FL* PCR product that did not respond to enoxacin treatment in any of the cell lines tested. To determine the specificity of this PCR product for *MdmX*, we performed siRNA-mediated MdmX knock-down in A375 and MCF7 cells, followed by the RT-PCR with primers 2. Results presented in S1B Fig show MdmX protein downregulation in response to siRNAs targeting *MdmX* in both cell lines and a substantial decrease in the *MdmX-FL* PCR product in both RT-PCR reactions, while there was no such change in the additional band, suggesting that it is a non-specific PCR product. Interestingly, in A375 cells we observed an increase in the amount of the *MdmX-S* product in siRNA-treated cells, indicating that siRNAs targeting the *MdmX* transcript might promote exon 6 skipping. This result was in line with a recent report suggesting that M*dmX* antisense oligonucleotides could induce exon 6 skipping [10].

To test the possibility that enoxacin treatment might not only modulate *MdmX* splicing but could also influence *MdmX* gene expression, we performed quantitative real-time PCR analysis in control and enoxacin-treated A375 cells (S1C Fig). Reverse transcriptions were performed using oligo(dT) and random hexamer primers to analyze both polyadenylated mRNAs and transcripts lacking poly(A). The difference between control and enoxacin-treated samples was not significant.

Next, we performed the *MdmX* splicing assay to compare the response of the three different cell lines overexpressing MdmX (A375, A2780, MCF7) to ciprofloxacin, another fluoroquinolone capable of promoting miRNA processing, and ofloxacin, a fluoroquinolone drug with reported low activity in the miRNA processing pathway [26]. Consistent with our hypothesis that the switch in *MdmX* splicing might represent a response of cancer cells overexpressing MdmX to changes in miRNA processing, ciprofloxacin induced *MdmX* exon 6 skipping in all three cell lines, while ofloxacin did not (Fig 4C).

**Fig 5:**
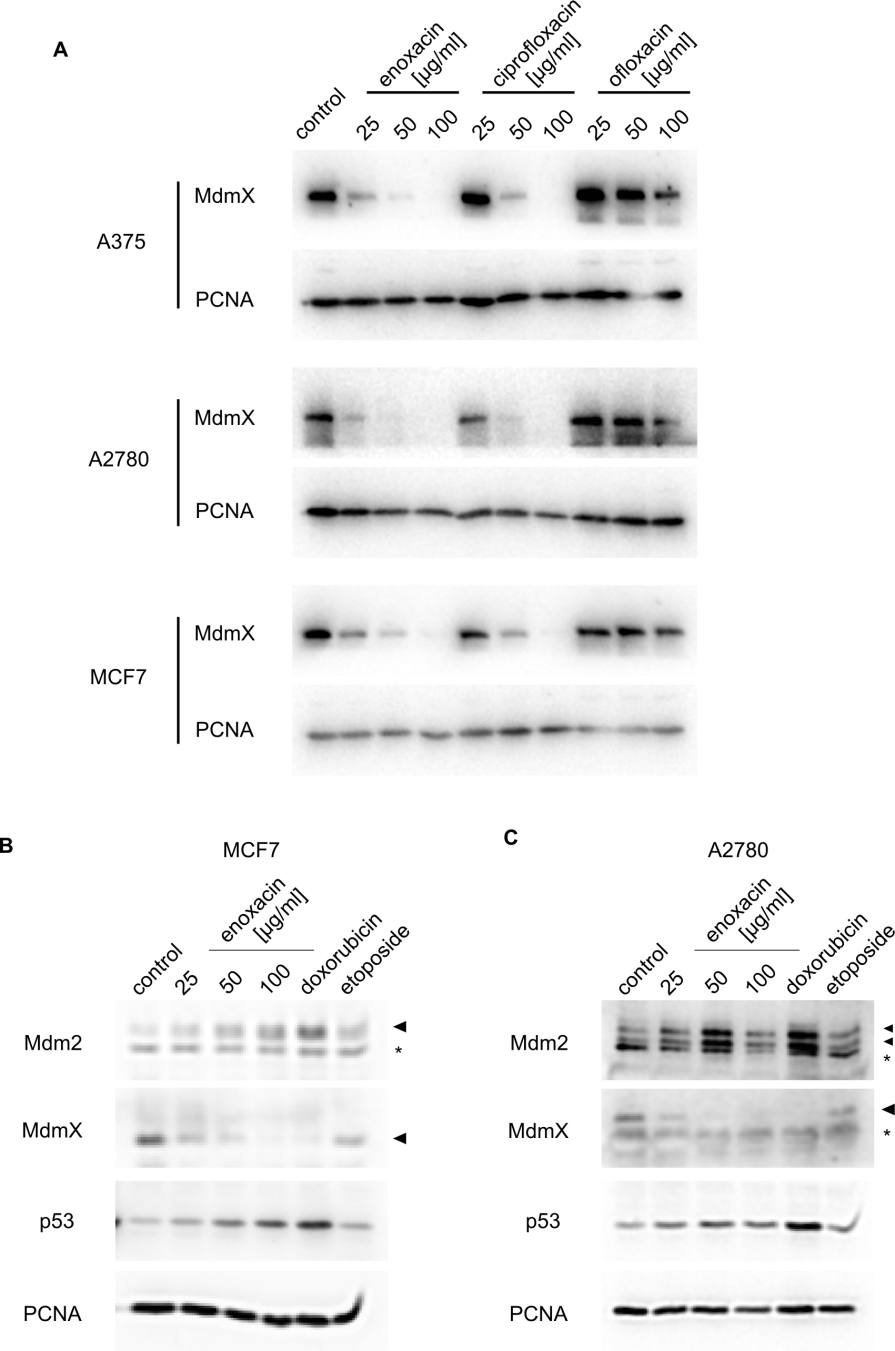
Enoxacin promotes MdmX downregulation in different cancer types. (**A**) Western blot analysis of changes in cellular MdmX protein levels induced by 24 h treatment with three fluoroquinolone drugs at indicated concentrations in A375 melanoma, A2780 ovarian carcinoma, and MCF7 breast carcinoma. PCNA served as a loading control. (**B**)(**C**) Western blot analysis of changes in cellular MdmX, Mdm2, and p53 protein levels induced by 24 h treatment with enoxacin and DNA damaging agents. PCNA served as a loading control. (B) MCF7, (C) A2780, * non-specific band.

Moreover, when we compared the response of the three different cancer lines overexpressing MdmX subjected to increasing concentrations of enoxacin, ciprofloxacin, and ofloxacin by Western blotting, we found that carcinoma cell lines responded like A375 melanoma cells. Enoxacin and ciprofloxacin very efficiently downregulated MdmX protein levels, while ofloxacin had only a weak effect on MdmX (Fig 5A). Like in A375, the response of A2780 and MCF7 cells to enoxacin was not associated with significant changes in Mdm2 splicing pattern and only small changes in p53 protein levels were detected (Fig 5B and 5C).

In the next set of experiments, we wanted to determine the role of the p53 status in the observed cellular responses to enoxacin. We started by comparing the effect of enoxacin on the growth of A375 and MCF7 and their derivatives MCF7-DDp53 and A375-DDp53 stably overexpressing a dominant-negative truncated mouse p53 protein [20,21]. A transient transfection of the p53-responsive luciferase construct into wild-type A375 and two A375 DDp53 clones (S2A Fig) and MCF7 plus two MCF7 DDp53 clones (S2B Fig), followed by enoxacin and doxorubicin treatments, confirmed that p53 activity was severely compromised in the DDp53-expressing A375 and MCF7 clones. Next, we analyzed the growth of the DDp53 cells in the presence of increasing concentrations of enoxacin using MTT assays. Enoxacin significantly inhibited the growth of A375 (S2C Fig) and MCF7 cells (S2D Fig), and the differences between the parental cell lines and DDp53 clones were not significant. These results suggested that other cellular pathways could be more important than p53 for determining the response of A375 and MCF7 cells to enoxacin. However, MdmX inhibition and p53 activation could still contribute to enoxacin activity in the context of other cancers overexpressing MdmX.

We also looked at the possibility that wild type p53 might be required for the switch in MdmX splicing to take place. For this, we chose two cell lines expressing low MdmX protein levels in which the p53 pathway is inhibited by other mechanisms. Human non-small cell lung carcinoma cells H1299 do not express the p53 protein, while the human melanoma cell line IPC298 expresses a mutant form of p53 (IARC TP53 Database). The growth of both cell lines was inhibited in the presence of enoxacin (S3B and S3C Figs). The analysis of *MdmX* exon 6 splicing did not show any difference between control and enoxacin-treated H1299 cells (S3A Fig). However, in IPC298 melanoma enoxacin promoted the switch between the *MdmX-FL* and *MdmX-S* isoforms, suggesting that the wild type p53 status might not be required for the switch in splicing to occur.

## Discussion

Small molecule fluoroquinolone antibiotics have a long history of relatively safe use in human medicine as antibacterial agents. In addition to that, later reports suggested that fluoroquinolones might exhibit specific biological activities also in mammalian cells. Enoxacin was identified in a chemical library screen to promote RNA interference by facilitating the interaction between pre-miRNAs and TARBP2, a RISC complex protein involved in miRNA maturation in mammalian cells [26]. Many miRNAs act as tumor suppressors and human cancers often exhibit global down-regulation of microRNA expression. Restoration of normal microRNA levels might, therefore, represent an attractive approach in cancer therapy [15–17,39]. Many microRNAs have been shown to be deregulated during melanoma development and progression [40,41] and recent research also hints at the possibility of a general decrease in miRNA-mediated silencing of gene expression in some melanomas due to the down-regulation of a key component of RISC, the RNA-induced silencing complex [42]. Enoxacin effectively restored miRNA processing in a panel of cancer cell lines from several common malignancies and had a TRBP-dependent cancer-specific inhibitory effect on cell growth both *in vitro* and in mouse xenografts [18,19]. The practical use of fluoroquinolones in cancer therapy might be limited by the fact that doses required for enhancing miRNA processing in mammalian cells are higher than those given to patients to treat bacterial infections. On the other hand, recent studies suggest that the active concentrations of fluoroquinolones can be achieved *in vivo* without major toxic side effects. For example, enoxacin inhibited the growth of xenografted human colorectal tumor cells in mice [18]. In a different study, the drug penetrated the blood-brain barrier, activated miRNA production in the frontal cortex, and decreased the proportion of rats exhibiting a depression-like disorder [43]. Our experiments suggest that enoxacin can inhibit the growth of human melanoma regardless of the type of mutation in the ERK MAP kinase pathway driving cell proliferation. This could be important because the metastatic form of melanoma remains incurable by current therapies, despite a significant progress over the last years in the development of targeted therapeutics such as vemurafenib (PLX4032) or dabrafenib, small molecule inhibitors of mutated BRAF kinase [44,45]. On the molecular level, we show that a switch in *MdmX* mRNA alternative splicing and down-regulation of MdmX protein levels, leading to non-genotoxic activation of p53-dependent transcription, could partly contribute to the antiproliferative action of fluoroquinolone antibiotics in melanoma cells expressing high levels of the *MdmX* oncogene. While we cannot exclude the possibility that enoxacin and ciprofloxacin could directly bind to the components of cellular splicing machinery and influence its activity, our data indicate that the ability of these drugs to modulate RNA interference might participate in the observed switch in *MdmX* splicing. Many splicing machinery genes were identified among the targets of miRNAs significantly dysregulated in response to enoxacin in A375 melanoma cells. We propose that they might collectively interfere with the splicing machinery, leading to a switch in *MdmX* mRNA alternative splicing, MdmX protein downregulation, and p53 activation, as reported in other studies using siRNAs and shRNAs to target individual components of the splicing apparatus [10,35]. In addition to melanoma, there are other types of cancer commonly retaining wild type *p53* gene and overexpressing MdmX, and the downregulation of *MdmX* expression in these tumors could also provide therapeutic benefit to patients. Therefore, it is important that the effect of fluoroquinolones on *MdmX* splicing was not limited to melanoma and that other types of cancer responded to enoxacin and ciprofloxacin by downregulating MdmX levels too. While millions of patients are prescribed fluoroquinolone antibiotics every year in the United States alone, there are some concerns about their safety, overuse, and potential adverse side effects to patients, especially children [46–48]. There are several reports suggesting genotoxicity of fluoroquinolones, esp. ciprofloxacin, in human cells *in vitro* but these drugs do not seem to be genotoxic *in vivo* [49,50]. We tested the possibility that enoxacin induced DNA damage in melanoma cells, as this might not only activate p53 independently of MdmX downregulation by inhibiting Mdm2 activity towards p53 but could also lead to changes in *MdmX* splicing [36,37]. In contrast to cells treated with DNA damaging agents, we did not see significant p53 stabilization in response to enoxacin treatment, especially after shorter treatments, that, however, already had an impact on MdmX protein levels. This result suggested that the drug is not inducing posttranslational modifications inhibiting the function of Mdm2 towards p53 that are characteristic of the cellular response to genotoxic stressors [51]. Moreover, the specific phosphorylation on serine 15 of human p53 was detected only in lysates of doxorubicin or etoposide treated cells, not in cells treated with enoxacin. Last, but not least, unlike the chemotherapeutic agents, enoxacin failed to induce any phosphorylation of histone H2AX on serine 139, a marker of double strand DNA breaks [52]. Together, these results speak against the possibility that enoxacin induced, at least in the concentrations tested, a significant amount of DNA damage.

Collectively, our data indicate that p53 activation might contribute to enoxacin activity against some human cancers and suggest that clinically approved fluoroquinolone drugs could potentially be repurposed as non-genotoxic activators of p53 in cancers cells overexpressing MdmX.

## Supporting information

S1 Fig (A) p53 activity in enoxacin-treated A375 cells stably transfected with pGL4.38 [luc2P/p53 RE/Hygro] luciferase construct. (B) siRNA-mediated knockdown of *MdmX* expression and its effect on the *MdmX* splicing assay using Primers 2. Western blotting for MdmX (left panel), gel electrophoresis of RT-PCR products (right panel). This result suggested that the additional band (marked with *) was a non-specific product of the PCR reaction not related to *MdmX*. (C) Real-time PCR analysis of the effect of enoxacin on MdmX gene expression. cDNA was obtained using oligo(dT) or random hexamer primers.
(TIFF)

S2 Fig Active p53 is not required for the inhibition of A375 and MCF7 cell growth by enoxacin. (A) A375/A375 DDp53, (B) MCF7/MCF7 DDp53. Analysis of p53 transcriptional activity in parental cells and clones overexpressing inhibitory mini protein p53DD. (C) A375/A375 DDp53, (D) MCF7/MCF7 DDp53. Analysis of cell growth inhibition in the presence of enoxacin by MTT assays (48 hours). Doxorubicin (0.5 μM) was used as a positive control. Results of three independent experiments are presented (means + SD). A significant difference between control and antibiotic-treated cells * P<0.05, ** P<0.01 (Student’s t-test, two-tailed).
(TIFF)

S3 Fig Enoxacin promotes *MdmX* alternative splicing in cells lacking functional p53. (A) Analysis of *MdmX* alternative splicing (exon 6 skipping) in H1299 and IPC298 cells treated for 24 hours with enoxacin (100 μg/ml). (B) H1299, (C) IPC298. Analysis of cell growth inhibition in the presence of enoxacin by MTT assays (48 hours). Doxorubicin (0.5 μM) was used as a positive control. Results of three independent experiments are presented (means + SD). A significant difference between control and antibiotic-treated cells * P<0.05, ** P<0.01 (Student’s t-test, two-tailed).
(TIFF)

S1 Table MicroRNAs significantly changing in A375 cells in response to enoxacin. List of miRNAs significantly up- and down-regulated in A375 melanoma in response to 24-hour treatment with enoxacin (p<0.05; three independent experiments).
(XLSX)

S2 Table Cellular pathways targeted by the miRNA dysregulated by enoxacin in A375 cells. (XLSX)

S3 Table Functional profile of 3924 miRNA target genes at Gene Ontology level (molecular functions, biological processes, cellular components). (XLSX)

S4 Table Gene set enrichment analysis of miRNA target genes. (XLSX)

S5 Table Potential miRNA target genes implicated in RNA splicing. (XLSX)

## References

[1] Vousden KH, Lane DP. P53 in Health and Disease. Nat Rev Mol Cell Biol. 2007;8: 275–83. doi: 10.1038/nrm2147 17380161 10.1038/nrm2147

[2] Wade M, Li YC, Wahl GM. MDM2, MDMX and p53 in oncogenesis and cancer therapy. Nat Rev Cancer. 2013;13: 83–96. doi: 10.1038/nrc3430 23303139 10.1038/nrc3430 PMC4161369

[3] Toledo F, Wahl GM. Regulating the p53 pathway: in vitro hypotheses, in vivo veritas. Nat Rev Cancer. 2006;6: 909–23. doi: 10.1038/nrc2012 17128209 10.1038/nrc2012

[4] Gembarska A, Luciani F, Fedele C, Russell EA, Dewaele M, Villar S, et al MDM4 is a key therapeutic target in cutaneous melanoma. Nat Med. 2012;18: 1239–1247. doi: 10.1038/nm.2863 22820643 10.1038/nm.2863 PMC3744207

[5] Laurie NA, Donovan SL, Shih C-S, Zhang J, Mills N, Fuller C, et al Inactivation of the p53 pathway in retinoblastoma. Nature. 2006;444: 61–6. doi: 10.1038/nature05194 17080083 10.1038/nature05194

[6] McEvoy J, Ulyanov A, Brennan R, Wu G, Pounds S, Zhang J, et al Analysis of MDM2 and MDM4 single nucleotide polymorphisms, mRNA splicing and protein expression in retinoblastoma. PLoS One. 2012;7: 1–11. doi: 10.1371/journal.pone.0042739 22916154 10.1371/journal.pone.0042739 PMC3423419

[7] Lam S, Lodder K, Teunisse AFAS, Rabelink MJWE, Schutte M, Jochemsen AG. Role of Mdm4 in drug sensitivity of breast cancer cells. Oncogene. 2010;29: 2415–26. doi: 10.1038/onc.2009.522 20140020 10.1038/onc.2009.522

[8] Yu Q, Li Y, Mu K, Li Z, Meng Q, Wu X, et al Amplification of Mdmx and overexpression of MDM2 contribute to mammary carcinogenesis by substituting for p53 mutations. Diagn Pathol. 2014;9: 71 doi: 10.1186/1746-1596-9-71 24667108 10.1186/1746-1596-9-71 PMC3986947

[9] Bo MD, Secchiero P, Degan M, Marconi D, Bomben R, Pozzato G, et al MDM4 (MDMX) is overexpressed in chronic lymphocytic leukaemia (CLL) and marks a subset of p53wild-type CLL with a poor cytotoxic response to Nutlin-3. British Journal of Haematology. 2010;150: 237–239. doi: 10.1111/j.1365-2141.2010.08185.x 20507307 10.1111/j.1365-2141.2010.08185.x

[10] Dewaele M, Tabaglio T, Willekens K, Bezzi M, Teo SX, Low DHP, et al Antisense oligonucleotide–mediated MDM4 exon 6 skipping impairs tumor growth. J Clin Invest. 2016;126: 68–84. doi: 10.1172/JCI82534 26595814 10.1172/JCI82534 PMC4701541

[11] Lenos K, Grawenda AM, Lodder K, Kuijjer ML, Teunisse AFAS, Repapi E, et al Alternate splicing of the p53 inhibitor HDMX offers a superior prognostic biomarker than p53 mutation in human cancer. Cancer Res. 2012;72: 4074–4084. doi: 10.1158/0008-5472.CAN-12-0215 22700878 10.1158/0008-5472.CAN-12-0215

[12] Liu L, Fan L, Fang C, Zou Z, Yang S, Zhang L, et al S-MDM4 mRNA overexpression indicates a poor prognosis and marks a potential therapeutic target in chronic lymphocytic leukemia. Cancer Sci. 2012;103: 2056–2063. doi: 10.1111/cas.12008 22937789 10.1111/cas.12008 PMC7659220

[13] Grawenda AM, Moller EK, Lam S, Repapi E, Teunisse AFAS, Alnas GIG, et al Interaction between p53 mutation and a somatic HDMX biomarker better defines metastatic potential in breast cancer. Cancer Res. 2015;75: 698–708. doi: 10.1158/0008-5472.CAN-14-2637 25649770 10.1158/0008-5472.CAN-14-2637

[14] Krol J, Loedige I, Filipowicz W. The widespread regulation of microRNA biogenesis, function and decay. Nat Rev Genet. 2010;11: 597–610. doi: 10.1038/nrg2843 20661255 10.1038/nrg2843

[15] Thomson JM, Newman M, Parker JS, Morin-Kensicki EM, Wright T, Hammond SM. Extensive post-transcriptional regulation of microRNAs and its implications for cancer. Genes Dev. 2006;20: 2202–2207. doi: 10.1101/gad.1444406 16882971 10.1101/gad.1444406 PMC1553203

[16] Lu J, Getz G, Miska EA, Alvarez-Saavedra E, Lamb J, Peck D, et al MicroRNA expression profiles classify human cancers. Nature. 2005;435: 834–838. doi: 10.1038/nature03702 15944708 10.1038/nature03702

[17] Blandino G, Fazi F, Donzelli S, Kedmi M, Sas-Chen A, Muti P, et al Tumor suppressor microRNAs: A novel non-coding alliance against cancer. FEBS Lett. Federation of European Biochemical Societies; 2014;588: 2639–2652. doi: 10.1016/j.febslet.2014.03.033 24681102 10.1016/j.febslet.2014.03.033

[18] Melo S, Villanueva A. Small molecule enoxacin is a cancer-specific growth inhibitor that acts by enhancing TAR RNA-binding protein 2-mediated microRNA processing. Proc Natl Acad Sci. 2011;108: 4394–4399. doi: 10.1073/pnas.1014720108 21368194 10.1073/pnas.1014720108 PMC3060242

[19] Sousa EJ, Graça I, Baptista T, Vieira FQ, Palmeira C, Henrique R, et al Enoxacin inhibits growth of prostate cancer cells and effectively restores microRNA processing. Epigenetics. 2013;8: 548–558. doi: 10.4161/epi.24519 23644875 10.4161/epi.24519 PMC3741225

[20] Kotala V, Uldrijan S, Horky M, Trbusek M, Strnad M, Vojtesek B. Potent induction of wild-type p53-dependent transcription in tumour cells by a synthetic inhibitor of cyclin-dependent kinases. Cell Mol Life Sci. 2001;58: 1333–9. doi: 10.1007/PL00000944 11577989 10.1007/PL00000944 PMC11337398

[21] Hammerova J, Uldrijan S, Taborská E, Slaninova I. Benzo[c]phenanthridine alkaloids exhibit strong anti-proliferative activity in malignant melanoma cells regardless of their p53 status. J Dermatol Sci. 2011;62: 22–35. doi: 10.1016/j.jdermsci.2011.01.006 21324654 10.1016/j.jdermsci.2011.01.006

[22] Bartel F, Schulz J, Böhnke A, Blümke K, Kappler M, Bache M, et al Significance of HDMX-S (or MDM4) mRNA splice variant overexpression and HDMX gene amplification on primary soft tissue sarcoma prognosis. Int J Cancer. 2005;117: 469–475. doi: 10.1002/ijc.21206 15906355 10.1002/ijc.21206

[23] Carvalho BS, Irizarry RA. A framework for oligonucleotide microarray preprocessing. Bioinformatics. 2010;26: 2363–2367. doi: 10.1093/bioinformatics/btq431 20688976 10.1093/bioinformatics/btq431 PMC2944196

[24] R Development Core Team. R: A language and environment for statistical computing R Foundation for Statistical Computing, Vienna, Austria URL http://www.R-project.org/. R Foundation for Statistical Computing, Vienna, Austria. 2013.

[25] Smyth GK. Limma: Linear Models for Microarray Data In: Gentleman R., Carey V., Dudoit S., Irizarry WH R., editor. Bioinformatics and Computational Biology Solutions Using R and Bioconductor. New York, NY: Springer New York; 2005 pp. 397–420. citeulike-article-id:5722720

[26] Shan G, Li Y, Zhang J, Li W, Szulwach KE, Duan R, et al A small molecule enhances RNA interference and promotes microRNA processing. Nat Biotechnol. 2008;26: 933–940. doi: 10.1038/nbt.1481 18641635 10.1038/nbt.1481 PMC2831467

[27] Pajak M, Simpson TI. miRNAtap: microRNA Targets—Aggregated Predictions. R package. 2015.

[28] Maragkakis M, Vergoulis T, Alexiou P, Reczko M, Plomaritou K, Gousis M, et al DIANA-microT Web server upgrade supports Fly and Worm miRNA target prediction and bibliographic miRNA to disease association. Nucleic Acids Res. 2011;39 doi: 10.1093/nar/gkr294 21551220 10.1093/nar/gkr294 PMC3125744

[29] Enright AJ, John B, Gaul U, Tuschl T, Sander C, Marks DS. MicroRNA targets in Drosophila. Genome Biol. 2003;5: R1 doi: 10.1186/gb-2003-5-1-r1 14709173 10.1186/gb-2003-5-1-r1 PMC395733

[30] Lall S, Grün D, Krek A, Chen K, Wang YL, Dewey CN, et al A genome-wide map of conserved MicroRNA targets in C. elegans. Curr Biol. 2006;16: 460–471. doi: 10.1016/j.cub.2006.01.050 16458514 10.1016/j.cub.2006.01.050

[31] Friedman RC, Farh KKH, Burge CB, Bartel DP. Most mammalian mRNAs are conserved targets of microRNAs. Genome Res. 2009;19: 92–105. doi: 10.1101/gr.082701.108 18955434 10.1101/gr.082701.108 PMC2612969

[32] Wong N, Wang X. miRDB: An online resource for microRNA target prediction and functional annotations. Nucleic Acids Res. 2015;43: D146–D152. doi: 10.1093/nar/gku1104 25378301 10.1093/nar/gku1104 PMC4383922

[33] Hoffman Y, Pilpel Y, Oren M. MicroRNAs and Alu elements in the p53-Mdm2-Mdm4 regulatory network. J Mol Cell Biol. 2014;6: 192–197. doi: 10.1093/jmcb/mju020 24868102 10.1093/jmcb/mju020 PMC4092252

[34] Chou C-H, Chang N-W, Shrestha S, Hsu S-D, Lin Y-L, Lee W-H, et al miRTarBase 2016: updates to the experimentally validated miRNA-target interactions database. Nucleic Acids Res. 2016;44: D239–D247. doi: 10.1093/nar/gkv1258 26590260 10.1093/nar/gkv1258 PMC4702890

[35] Allende-Vega N, Dayal S, Agarwala U, Sparks A, Bourdon J-C, Saville MK. p53 is activated in response to disruption of the pre-mRNA splicing machinery. Oncogene. Nature Publishing Group; 2013;32: 1–14. doi: 10.1038/onc.2012.38 22349816 10.1038/onc.2012.38

[36] Chandler DS, Singh RK, Caldwell LC, Bitler JL, Lozano G. Genotoxic stress induces coordinately regulated alternative splicing of the p53 modulators MDM2 and MDM4. Cancer Res. 2006;66: 9502–9508. doi: 10.1158/0008-5472.CAN-05-4271 17018606 10.1158/0008-5472.CAN-05-4271

[37] Dutertre M, Sanchez G, De Cian M-C, Barbier J, Dardenne E, Gratadou L, et al Cotranscriptional exon skipping in the genotoxic stress response. Nat Struct Mol Biol. 2010;17: 1358–1366. doi: 10.1038/nsmb.1912 20972445 10.1038/nsmb.1912

[38] Jacob AG, Singh RK, Comiskey DF, Rouhier MF, Mohammad F, Bebee TW, et al Stress-induced alternative splice forms of MDM2 and MDMX modulate the p53-pathway in distinct ways. PLoS One. 2014;9 doi: 10.1371/journal.pone.0104444 25105592 10.1371/journal.pone.0104444 PMC4126728

[39] Adams BD, Kasinski AL, Slack FJ. Aberrant Regulation and Function of MicroRNAs in Cancer. Curr Biol. Elsevier Ltd; 2014;24: R762–R776. doi: 10.1016/j.cub.2014.06.043 25137592 10.1016/j.cub.2014.06.043 PMC4177046

[40] Philippidou D, Schmitt M, Moser D, Margue C, Nazarov P V., Muller A, et al Signatures of MicroRNAs and selected MicroRNA target genes in human melanoma. Cancer Res. 2010;70: 4163–4173. doi: 10.1158/0008-5472.CAN-09-4512 20442294 10.1158/0008-5472.CAN-09-4512

[41] Caramuta S, Egyházi S, Rodolfo M, Witten D, Hansson J, Larsson C, et al MicroRNA expression profiles associated with mutational status and survival in malignant melanoma. J Invest Dermatol. 2010;130: 2062–2070. doi: 10.1038/jid.2010.63 20357817 10.1038/jid.2010.63

[42] Völler D, Reinders J, Meister G, Bosserhoff A-K. Strong reduction of AGO2 expression in melanoma and cellular consequences. Br J Cancer. 2013;109: 3116–24. doi: 10.1038/bjc.2013.646 24169347 10.1038/bjc.2013.646 PMC3859937

[43] Smalheiser NR, Zhang H, Dwivedi Y. Enoxacin elevates microRNA levels in rat frontal cortex and prevents learned helplessness. Front Psychiatry. 2014;5: 1–6. doi: 10.3389/fpsyt.2014.00001 24575053 10.3389/fpsyt.2014.00006 PMC3918929

[44] Sosman JA, Kim KB, Schuchter L, Gonzalez R, Pavlick AC, Weber JS, et al Survival in BRAF V600-mutant advanced melanoma treated with vemurafenib. N Engl J Med. 2012;366: 707–14. doi: 10.1056/NEJMoa1112302 22356324 10.1056/NEJMoa1112302 PMC3724515

[45] Hauschild A, Grob JJ, Demidov LV, Jouary T, Gutzmer R, Millward M, et al Dabrafenib in BRAF-mutated metastatic melanoma: A multicentre, open-label, phase 3 randomised controlled trial. Lancet. 2012;380: 358–365. doi: 10.1016/S0140-6736(12)60868-X 22735384 10.1016/S0140-6736(12)60868-X

[46] Almalki ZS, Alahmari AK, Guo JJ, Cavanaugh TM. Off-label use of oral fluoroquinolone antibiotics in outpatient settings in the United States, 2006 to 2012. Pharmacoepidemiol Drug Saf. 2016;25: 1042–1051. doi: 10.1002/pds.4021 27133913 10.1002/pds.4021

[47] Bourgeois T, Delezoide AL, Zhao W, Guimiot F, Adle-Biassette H, Durand E, et al Safety study of Ciprofloxacin in newborn mice. Regul Toxicol Pharmacol. 2016;74: 161–169. doi: 10.1016/j.yrtph.2015.11.002 26627140 10.1016/j.yrtph.2015.11.002

[48] Patel K, Goldman JL. Safety Concerns Surrounding Quinolone Use in Children. J Clin Pharmacol. 2016;56: 1060–1075. doi: 10.1002/jcph.715 26865283 10.1002/jcph.715 PMC4994191

[49] Itoh T, Mitsumori K, Kawaguchi S, Sasaki YF. Genotoxic potential of quinolone antimicrobials in the in vitro comet assay and micronucleus test. Mutat Res—Genet Toxicol Environ Mutagen. 2006;603: 135–144. doi: 10.1016/j.mrgentox.2005.11.003 16384725 10.1016/j.mrgentox.2005.11.003

[50] Herbold BA, Brendler-Schwaab SY, Ahr HJ. Ciprofloxacin: In vivo genotoxicity studies. Mutat Res—Genet Toxicol Environ Mutagen. 2001;498: 193–205. doi: 10.1016/S1383-5718(01)00275-3 10.1016/s1383-5718(01)00275-3 11673084

[51] Carr MI, Jones SN. Regulation of the Mdm2-p53 signaling axis in the DNA damage response and tumorigenesis. Transl Cancer Res. 2016;5: 707–724. doi: 10.21037/tcr.2016.11.75 28690977 10.21037/tcr.2016.11.75 PMC5501481

[52] Rogakou EP, Pilch DR, Orr AH, Ivanova VS, Bonner WM. Double-stranded Breaks Induce Histone H2AX phosphorylation on Serine 139. J Biol Chem. 1998;273: 5858–5868. doi: 10.1074/jbc.273.10.5858 9488723 10.1074/jbc.273.10.5858

