# Supplementary figures and images for: A small molecule drug promoting miRNA processing induces alternative splicing of MdmX transcript and rescues p53 activity in human cancer cells overexpressing MdmX protein
Source: PLoS One. 2017 Oct 3;12(10):e0185801. doi: 10.1371/journal.pone.0185801 (PMC5626491; doi:10.1371/journal.pone.0185801)

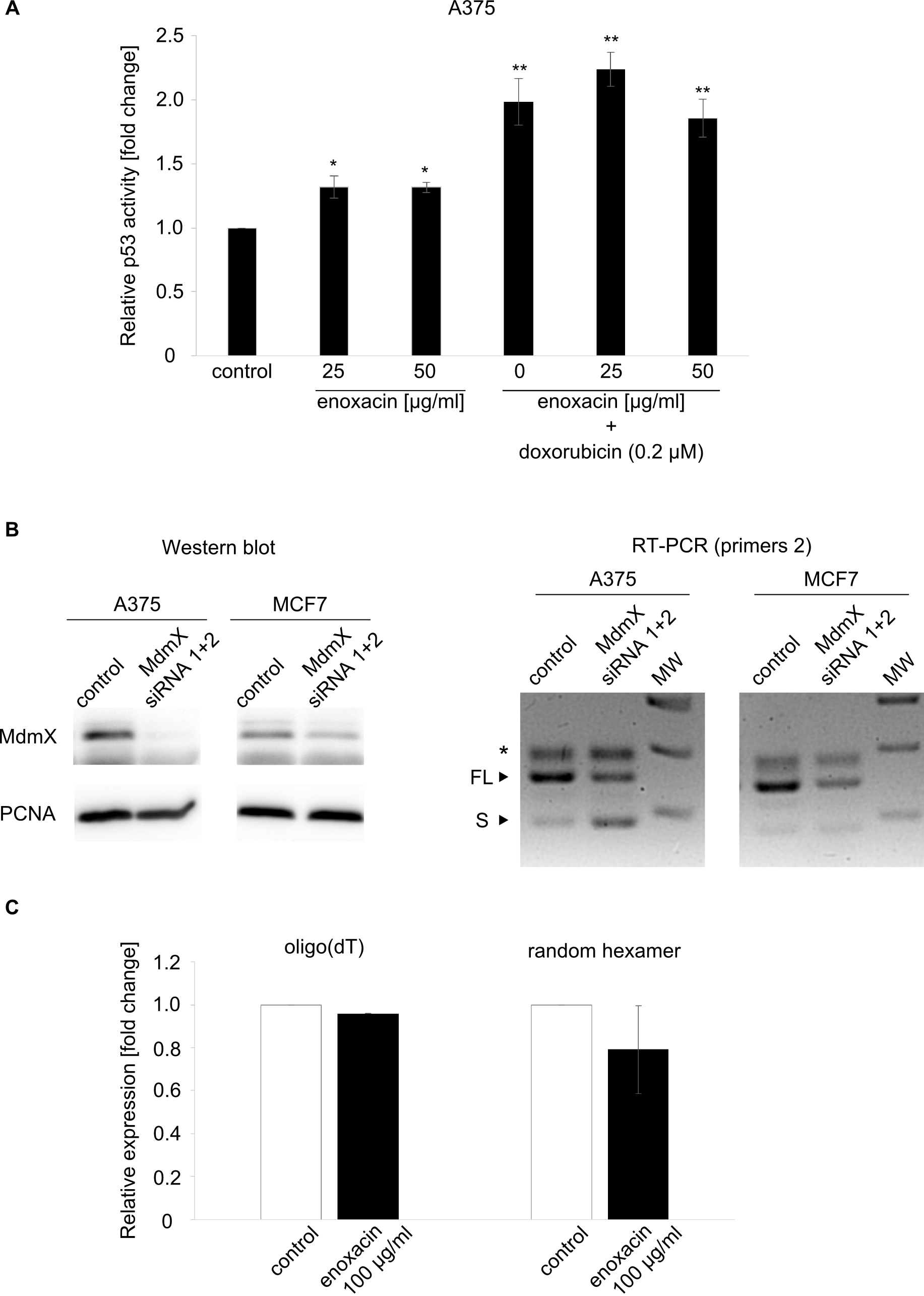

Supplement: S1 Fig — (A) p53 activity in enoxacin-treated A375 cells stably transfected with pGL4.38 [luc2P/p53 RE/Hygro] luciferase construct. (B) siRNA-mediated knockdown of MdmX expression and its effect on the MdmX splicing assay using Primers 2. Western blotting for MdmX (left panel), gel electrophoresis of RT-PCR products (right panel). This result suggested that the additional band (marked with *) was a non-specific product of the PCR reaction not related to MdmX. (C) Real-time PCR analysis of the effect of enoxacin on MdmX gene expression. cDNA was obtained using oligo(dT) or random hexamer primers. (TIFF) [file pone.0185801.s001.tiff]

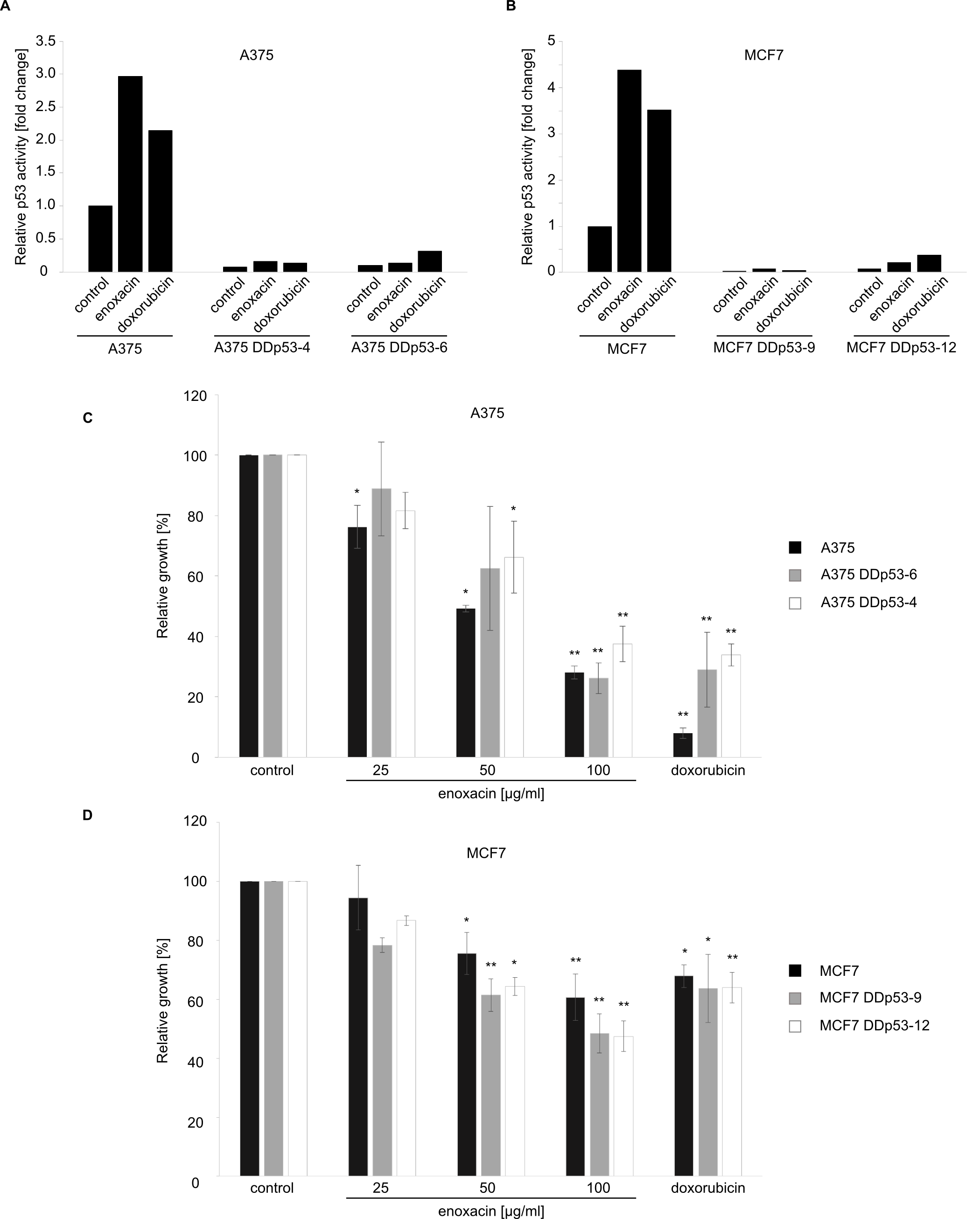

Supplement: S2 Fig — (A) A375/A375 DDp53, (B) MCF7/MCF7 DDp53. Analysis of p53 transcriptional activity in parental cells and clones overexpressing inhibitory mini protein p53DD. (C) A375/A375 DDp53, (D) MCF7/MCF7 DDp53. Analysis of cell growth inhibition in the presence of enoxacin by MTT assays (48 hours). Doxorubicin (0.5 μM) was used as a positive control. Results of three independent experiments are presented (means + SD). A significant difference between control and antibiotic-treated cells * P<0.05, ** P<0.01 (Student’s t-test, two-tailed). (TIFF) [file pone.0185801.s002.tiff]

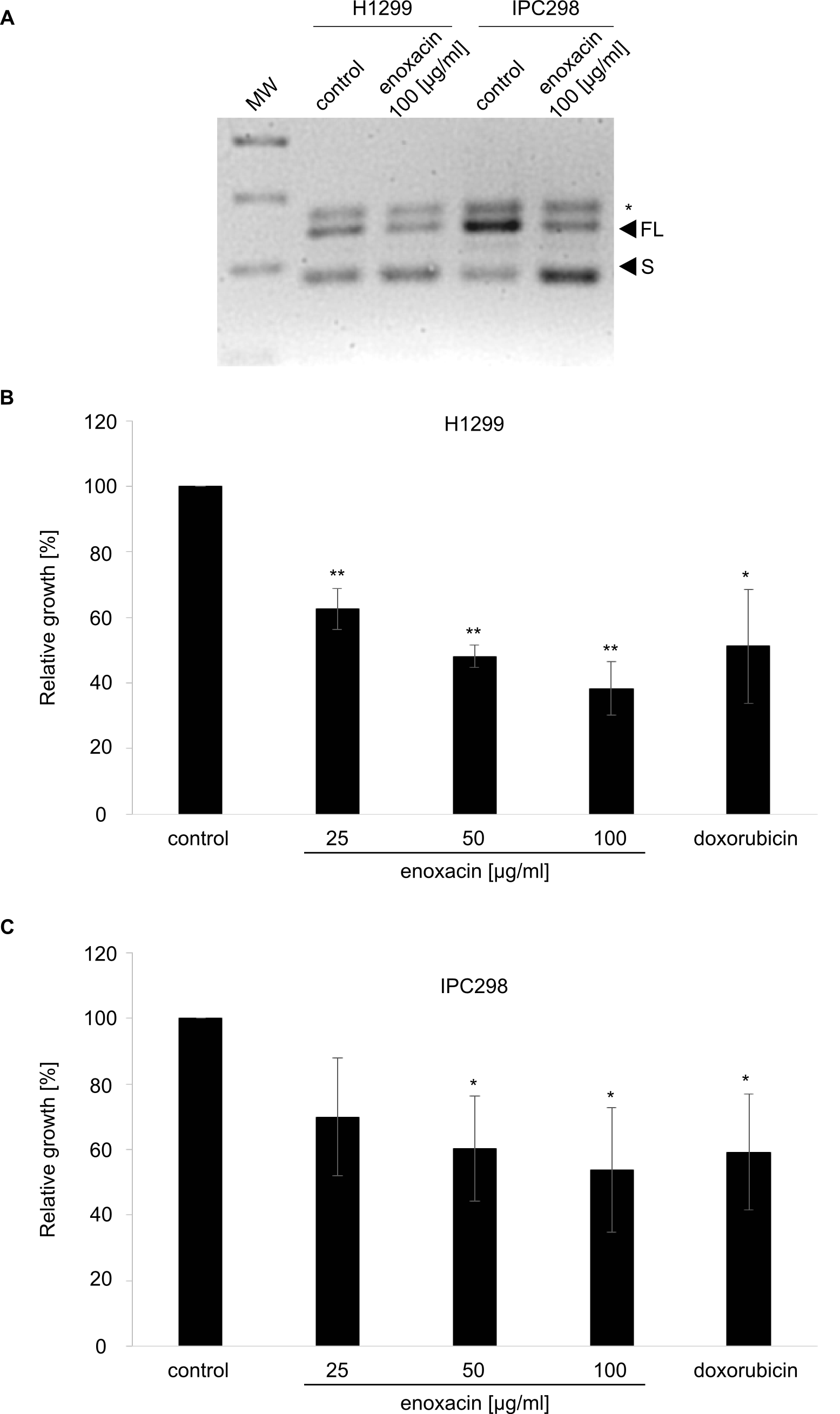

Supplement: S3 Fig — (A) Analysis of MdmX alternative splicing (exon 6 skipping) in H1299 and IPC298 cells treated for 24 hours with enoxacin (100 μg/ml). (B) H1299, (C) IPC298. Analysis of cell growth inhibition in the presence of enoxacin by MTT assays (48 hours). Doxorubicin (0.5 μM) was used as a positive control. Results of three independent experiments are presented (means + SD). A significant difference between control and antibiotic-treated cells * P<0.05, ** P<0.01 (Student’s t-test, two-tailed). (TIFF) [file pone.0185801.s003.tiff]
